# White Matter Integrity Supports BOLD Signal Variability and Cognitive Performance in the Aging Human Brain

**DOI:** 10.1371/journal.pone.0120315

**Published:** 2015-04-08

**Authors:** Agnieszka Z. Burzynska, Chelsea N. Wong, Michelle W. Voss, Gillian E. Cooke, Edward McAuley, Arthur F. Kramer

**Affiliations:** 1 The Beckman Institute for Advanced Science and Technology at the University of Illinois, 405 N. Mathews Ave, Urbana, IL, 61801, United States of America; 2 Department of Psychology, University of Iowa, E11 Seashore Hall, Iowa City, IA, 52242–1407, United States of America; 3 Department of Kinesiology and Community Health, University of Illinois, 906 S. Goodwin Ave. Urbana, IL, 61801, United States of America; University Of Cambridge, UNITED KINGDOM

## Abstract

Decline in cognitive performance in old age is linked to both suboptimal neural processing in grey matter (GM) and reduced integrity of white matter (WM), but the whole-brain structure-function-cognition associations remain poorly understood. Here we apply a novel measure of GM processing–moment-to-moment variability in the blood oxygenation level-dependent signal (SD_BOLD_)—to study the associations between GM function during resting state, performance on four main cognitive domains (i.e., fluid intelligence, perceptual speed, episodic memory, vocabulary), and WM microstructural integrity in 91 healthy older adults (aged 60-80 years). We modeled the relations between whole-GM SD_BOLD_ with cognitive performance using multivariate partial least squares analysis. We found that greater SD_BOLD_ was associated with better fluid abilities and memory. Most of regions showing behaviorally relevant SD_BOLD_ (e.g., precuneus and insula) were localized to inter- or intra-network “hubs” that connect and integrate segregated functional domains in the brain. Our results suggest that optimal dynamic range of neural processing in hub regions may support cognitive operations that specifically rely on the most flexible neural processing and complex cross-talk between different brain networks. Finally, we demonstrated that older adults with greater WM integrity in all major WM tracts had also greater SD_BOLD_ and better performance on tests of memory and fluid abilities. We conclude that SD_BOLD_ is a promising functional neural correlate of individual differences in cognition in healthy older adults and is supported by overall WM integrity.

## Introduction

Cognitive performance, such memory, reasoning, perceptual speed, and maintenance of semantic knowledge, relies on the neural processing in grey matter (GM) and the integrity of white matter (WM). Many neuroimaging studies attempt to link age-related differences in cognitive performance with either blood-oxygenation level dependent (BOLD) signal magnitude and localization [1,2] or WM integrity [3]. However, designs and results of the functional studies are region-, and cognitive task-specific, and therefore yield mixed results. As a consequence, combining whole-brain GM function with WM structure in aging remains a challenge and has been rarely attempted. Clearly, a more general functional measure would be useful in linking GM processing with WM integrity and individual differences in cognition in aging.

Recently, measuring the variability in the BOLD signal (SD_BOLD_) has emerged as a novel frontier in mapping human brain function in aging [4,5]. The brain–a dynamic system that undergoes spontaneous or external stimuli-driven moment-to-moment reconfigurations [6–11] –is inherently variable [12]. Older adults were shown to have reduced SD_BOLD_ in many GM regions compared to younger adults [13], and SD_BOLD_ was associated with faster and more consistent performance [14]. These positive associations between SD_BOLD_ and cognition in aging, however, are based only on performance on a perceptual matching task (instantaneous match-to-sample, attentional cueing, and delayed match-to-sample [14]). Here we address the yet unexplored questions: Can the link between higher SD_BOLD_ and cognitive performance be extended to other cognitive abilities known to decline with age, such as reasoning, speed, and episodic memory [15,16]? Is there a general pattern of higher SD_BOLD_ that supports cognitive functioning across cognitive domains or are patterns of SD_BOLD_ optimal for a specific cognitive function? Does WM integrity support behaviorally relevant SD_BOLD_ in the aging brain?

We collected resting-state fMRI and diffusion images as well as well-normed laboratory measures of fluid intelligence, perceptual speed, episodic memory, and vocabulary [17–20] from 104 healthy participants (60–80 years). Previous research showed that resting-state and task-related signal amplitude fluctuations are linearly related across subjects and voxels and may be governed by same neuronal and physiological mechanism [21]. Therefore, our approach of using resting-state signal minimizes the effect of task on SD_BOLD_, allowing SD_BOLD_ to be related to a broad range of cognitive abilities. We predicted greater SD_BOLD_, especially in hub regions highly connected within brain networks [22,23], to be related to better fluid abilities and memory, as they require more moment-to-moment adaptability in brain network utilization (e.g. association formation, mental rotation). We expected SD_BOLD_ to have a weaker relationship with vocabulary knowledge and perceptual speed, relying on rather stereotypical responses and semantic retrieval. We modeled the relations between whole-GM SD_BOLD_ with cognitive performance using multivariate partial least squares analysis (PLS; [24]). Finally, as greater WM integrity, measured as fractional anisotropy (FA), predicts higher cognitive performance and GM processing efficiency in older adults [3,25–27], we proposed WM integrity as a candidate structural correlate of the behaviorally relevant SD_BOLD_ in the aging brain.

We found that greater SD_BOLD_ was associated with better fluid abilities and memory, and this behaviorally relevant SD_BOLD_ was associated with WM integrity.

## Methods

### Participants

A University of Illinois Institutional Review Board approved the study, and written informed consent was obtained from all participants and the study was performed in accordance with the 1964 Declaration of Helsinki. Participants received financial reimbursement. We collected MRI and behavioral data from 111 community-dwelling healthy older adults (37 males). The sample contained more females because fewer older males met the above inclusion criteria or showed willingness to participate in the study. Eligible participants met the following criteria: (1) were between the ages of 60 and 79 years old, (2) were free from psychiatric and neurological illness and had no history of stroke or transient ischemic attack, (3) scored ≥ 27 on the Mini-Mental State Exam (MMSE) and >21 on a Telephone Interview of Cognitive Status (TICS-M) questionnaire, (4) scored < 10 on the geriatric depression scale (GDS-15), (5) scored ≥ 75% right-handedness on the Edinburgh Handedness Questionnaire, (6) demonstrated normal or corrected-to-normal vision of at least 20/40 and no color blindness, (7) were cleared for suitability in the MRI environment, that is, no metallic implants that could interfere with the magnetic field or cause injury, no claustrophobia, and no history of head trauma. The participants were a pre-intervention cross-sectional subsample from an on-going randomized controlled exercise trial (“Influence of Fitness on Brain and Cognition II” at ClinicalTrials.gov, clinical study identifier NCT01472744), from whom good quality anatomical and resting state functional MRI (see section 2.4 and 2.6) was available.

### Cognitive assessment and analysis

We administered a cognitive battery as described in the Virginia Cognitive Aging Project [17–20] to measure latent constructs of fluid intelligence, perceptual speed, episodic memory, and vocabulary (for more details on each task see Table 1). The computer-based tasks were programmed in E-prime version 1.1 (Psychology Software Tools, Pittsburgh, PA) and administered on computers with 17” cathode ray tube monitors.

**Table 1:** Cognitive battery and the result of dimensionality reduction with PCA.

Task	Construct	Description	Administration	Source	Fluid abilities	Perceptual Speed	Memory	Vocabulary
Matrix reasoning	Fluid intelligence	Select pattern that best completes the missing cell in a matrix	Computer-based	[77]	.628	–	–	.418
Shipley abstraction	Fluid intelligence	Determine the letters, words, or numbers that best complete a progressive sequence	Paper-pencil	[78]	.525	–	–	.564
Letter sets	Fluid intelligence	Identify which of five groups of letters is different from the others	Computer-based	[79]	.346	.410	–	.575
Spatial relations	Spatial reasoning	Determine which three dimensional object could be constructed by folding the two dimensional object	Computer-based	[80]	.788	–	–	–
Paper folding	Spatial reasoning	Determine the pattern of holes that would result from a sequence of folds and a punch through folded paper	Computer-based	[79]	.856	–	–	–
Form boards	Spatial reasoning	Determine shapes needed to fill in a space	Computer-based	[79]	.725	–	–	–
Digit symbol	Perceptual speed	Use a code table to write the correct symbol below each digit	Paper-pencil	[81]	–	.756	–	–
Letter/pattern comparison	Perceptual speed	Same or different comparison of pairs of letter strings/patterns	Paper-pencil	[82]	–/.346	.845/.797	–	–
Logical memory	Episodic memory	Recall as many idea units as possible from three stories	Computer-based/paper-pencil	[83]	–	–	.752	.319
Free recall	Episodic memory	Recall as many words as possible across four word trial lists	Computer-based/ paper-pencil	[83]	–	–	.789	–
Paired associates	Episodic memory	Recall the second words from word pairs	Computer-based/ paper-pencil	[84]	–	–	.787	–
WAIS vocab.	Vocabulary	Define words out loud	Experimenter/ paper-pencil	[81]	–	–	–	.778
Picture vocab.	Vocabulary	Name the objects presented	Experimenter/ paper-pencil	[85]	.383	–	–	.720
Synonym/ antonym	Vocabulary	Choose the word most similar/opposite in meaning to the target	Computer-based	[86]	–	–	–	.876/.857

Note. Columns 6–9: Standardized component loadings from a 4-factor PCA extraction. For clarity, only loadings above 0.30 are displayed. Rotation method: varimax with Kaiser normalization. Rotation converged in 6 iterations. Pairwise exclusion was performed.

To obtain components representing the four cognitive constructs and to confirm the validity of task structure as presented in [20], we performed principal component analysis (PCA) with varimax rotation. Individual scores on each of the 16 tasks were first screened for outliers and winsorized (maximum 3 cases out of 91 (<3%) were adjusted per variable). The resulting constructs are presented in Table 1 and the component scores were saved as variables.

Some participants did not complete all tasks in the cognitive battery, which resulted in a final sample of 91 participants (29 males, age range 60–78, M_age_ = 65 ± 4 years, years of education 12–26, M_edu_ = 17 ± 4 years).

### MRI acquisition

We acquired all images during a single session on a 3T Siemens Trio Tim system with 45 mT/m gradients and 200 T/m/sec slew rates (Siemens, Erlangen, Germany). T2*-weighted resting state images were acquired with fast echo-planar imaging (EPI) sequence with Blood Oxygenation Level Dependent (BOLD) contrast (6min, TR = 2s, TE = 25ms, flip angle = 80 degrees, 3.4 x3.4 mm^2^ in-plane resolution, 35 4mm-thick slices acquired in ascending order, Grappa acceleration factor = 2, 64 × 64 matrix). The participants were instructed to lay still with eyes closed. Additionally, gradient field maps were acquired to account for geometric distortions caused by magnetic field inhomogeneity [28]. The gradient field map was collected as 35, 4mm-thick slices, 3.4 x 3.4 mm^2^ in-plane resolution, TR = 700ms, TE = 10ms, and flip angle = 35 degrees.

High-resolution structural MR scans were acquired using a 3D MPRAGE T1-weighted sequence (TR = 1900 ms; TE = 2.32 ms; TI: 900 ms; flip angle = 9°; matrix = 256 × 256; FOV = 230mm; 192 slices; resolution = 0.9 × 0.9 × 0.9 mm; GRAPPA acceleration factor 2) and used as an intermediate step in registration of functional images to standard MNI space.

DTI images were acquired with a twice-refocused spin echo single-shot Echo Planar Imaging sequence [29] to minimize eddy current-induced image distortions. The protocol consisted of a set of 30 non-collinear diffusion-weighted acquisitions with b-value = 1000s/mm^2^ and two T2-weighted b-value = 0 s/mm^2^ acquisitions, repeated two times (TR/TE = 5500/98 ms, 128 x 128 matrix, 1.7x1.7 mm^2^ in-plane resolution, FA = 90, GRAPPA acceleration factor 2, and bandwidth of 1698 Hz/Px, comprising 40 3-mm-thick slices). Resting state and DTI images were obtained parallel to the anterior-posterior commissure plane with no interslice gap.

### BOLD variability (SD_BOLD_) calculation

Data preprocessing was carried out using FSL v5.0.1 (FMRIB's Software Library, http://www.fmrib.ox.ac.uk/fsl; [30]). The preprocessing included high pass filtering (> 0.008Hz), slice timing correction, rigid body motion correction using MCFLRT [31], and removal of non-brain tissue with the Brain Extraction Tool [32]. Data from all subjects was screened for motion and all participants moved within a voxel dimension (< 4mm). Functional images of each participant were aligned to the standard stereotaxic space of the MNI 152 T1 2mm^3^ template supplied in FSL in a three-step procedure. To improve the registration between the participant’s functional and anatomical images we utilized the gradient field map data. First, the gradient field map was unwrapped via PRELUDE [33], then geometric distortions in the EPI-related images due to local magnetic inhomogeneity differences were compensated for with the use of gradient field map data via FUGUE within FSL [33]. Eleven out of 91 participants had missing field map images. Second, each participant’s low-resolution functional images were aligned with their high-resolution T1-weighted anatomical images using the Boundary-Based Registration in FSL [34]. Third, the anatomical images were aligned to MNI 152T1 2mm^3^ template using 12 degrees of freedom affine linear registration [31].

Next, as recommended by [13], we used Multivariate Exploratory Linear Optimized Decomposition into Independent Components (MELODIC v3.10) tool in FSL [35] to decompose the 4D fMRI time series into spatial and temporal components. AZB together with Chanheng He and CNW identified artifact components for each subject using the criteria outlined in [36] based on the spatial pattern, time course, and power spectrum properties that were characteristic of physiological noise, motion, and scanner-related artifacts. The artifactual components were regressed out from the time series yielding the post-ICA ‘cleaned’ data. This post-ICA functional data as well as the six motion parameters outputted earlier by motion correction were bandpass filtered to restrict the frequencies in our data to. 008 < *f* <. 1 Hz [37]. Next, we extracted mean time series from two nuisance regions of interest (deep temporal white matter, bilateral lateral ventricles) in the post-ICA filtered data. The goal of including these two nuisance regressors is to remove residual cardiorespiratory physiological noise that would be captured by signal changes in the white matter and ventricles [38–41] and was not removed by the ICA cleanup. The two nuisance regressors (timeseries from white matter and ventricles) were regressed out using the general linear model with FEAT 6.00 (FMRI Expert Analysis Tool; http://www.fmrib.ox.ac.uk/analysis/research/feat/). Finally, we calculated the standard deviation (SD_BOLD_) across the whole timeseries for each voxel and smoothed the images with a 6mm Gaussian kernel. The resulting SD_BOLD_ maps were upsampled to MNI space using the registration steps described above. To restrict all multivariate analyses to the GM, we masked the SD_BOLD_ maps with the grey matter tissue prior provided in FSL, thresholded at probability > 0.37. The intermediate outcomes of all the above procedures were carefully inspected by AZB and CNW.

### PLS multivariate analysis of relations among SD_BOLD_, cognitive performance and fitness

First, we made sure that all behavioral variables were normally distributed and any outliers (> 2.5 SD) were accounted for by winsorizing, where not more than 2 cases were corrected per variable (2%).

The behavioral PLS analysis [42,43] begins with a correlation matrix (CORR) between our variables of interest (four cognitive constructs) and each voxel’s signal (SD_BOLD_); correlations are calculated across subjects. Then, this CORR matrix is decomposed via singular value decomposition (SVD): SVD_CORR_ = *USV*’. This decomposition produces a left singular vector of behavioral weights (*U*), a right singular vector of SD_BOLD_ weights (*V*), and a diagonal matrix of singular values (*S*). In other words, this analysis produces orthogonal latent variables (LVs) that optimally represent relations between behavior and SD_BOLD_ in grey matter voxels. Each LV contains a spatial pattern depicting the brain regions where the SD_BOLD_ shows the strongest relation to behavior. Each brain weight (in *V*) is proportional to the correlation between behavior and SD_BOLD_ in all of the tracts. To obtain a summary measure of each participant’s expression of a particular LV pattern, we calculated within-person “brain scores” by multiplying each voxel (*i*)’s weight (*V*) from each LV (*j*) produced from the SVD in equation (1) by the SD_BOLD_ value in that voxel for person (*k*), and summing over all (*n*) brain voxels: $\sum_{i\;=\;1}^{n}V_{ij}{SD\;}_{ik}$. Thus, in a single measure, a brain score indicates the degree to which a subject expresses the multivariate spatial pattern captured by a given behavior-driven latent variable. Significance of detected relations between multivariate spatial patterns and cognitive performance was assessed using 1000 permutation tests of the singular value corresponding to each LV. A subsequent bootstrapping procedure revealed the robustness of voxel saliences across 1000 bootstrapped resamples of our data [44]. By dividing each voxel’s mean salience by its bootstrapped standard error, we obtained “bootstrap ratios” as normalized estimates of robustness. We thresholded bootstrap ratios at a value of ≥ 3.00, which approximates a 99% confidence interval and corresponds to *p*-value of <.001.

### DTI analysis

DTI allows inferences about WM microstructure in vivo by quantifying the magnitude and directionality of diffusion of water within a tissue [45]. Visual checks were performed on every volume of the raw data of every participant by AZB. Sixty-six participants had good quality DTI data. In one dataset, one volume with the corresponding b-vectors and b-values was deleted from the dataset before processing due to artifact. Next, DTI data were processed using the FSL Diffusion Toolbox v.3.0 (FDT: http://www.fmrib.ox.ac.uk/fsl) in a standard multistep procedure, including: (a) motion and eddy current correction of the images and corresponding b-vectors, (b) removal of the skull and non-brain tissue using the Brain Extraction Tool [32], and (c) voxel-by-voxel calculation of the diffusion tensors. Using the diffusion tensor information, FA maps were computed using DTIFit within the FDT. All motion- and eddy-current outputs, as well as FA images were visually inspected.

We used TBSS [46,47], a toolbox within FSL v5.0.1, to create a representation of main WM tracts common to all subjects (WM “skeleton”). This included: (a) nonlinear alignment of each participant’s FA volume to the 1 x 1 x 1 mm^3^ standard Montreal Neurological Institute (MNI152) space via the FMRIB58_FA template using the FMRIB’s Nonlinear Registration Tool (FNIRT, [48]; http://www.doc.ic.ac.uk/~dr/software), (b) calculation of the mean of all aligned FA images, (c) creation of the WM “skeleton” by perpendicular non-maximum-suppression of the mean FA image and setting the FA threshold to 0.25, and (d) perpendicular projection of the highest FA value (local center of the tract) onto the skeleton, separately for each subject. The outputs of all the above processing steps were carefully inspected by AZB. Given that SD_BOLD_ is a relatively new way to asses brain function and its structural brain correlates are not yet understood, we did not make any regional predictions and used a global FA measure, obtained by averaging FA over the whole skeleton for each participant.

### Post hoc statistical analyses

All statistical analyses were performed using SPSS (v.16, SPSS Inc., Chicago, IL, USA). We used multiple step-wise linear regressions (with chronological age and gender) to investigate the relationships between brains scores from SD_BOLD_-cognition and global FA. Two participants brain scores had outlier values > 2.5 SD and their values were winsorized, which did not change the results and was used for display purposes.

The demographic data, FA values, behavioral scores, and brain scores are available in S1 Dataset.

## Results

### Correlations between cognitive performance and SD_BOLD_

To investigate the relationships between SD_BOLD_ and performance on four main cognitive domains, we first performed principal component analysis (PCA) on 16 tasks from Table 1 to reduce their dimensionality. We replicated the findings of the Salthouse studies [17–20] by obtaining the four expected components of fluid intelligence, perceptual speed, episodic memory, and vocabulary (Table 1). Only speed (r = -.33 p = .002) and memory (r = -31, p = .003) components were negatively related to age, whereas fluid abilities (r = -.18 p = .098) and vocabulary (r = .11 p = .320) were not.

Next, to identify multivariate across-subject patterns of relations between SD_BOLD_ at rest in the entire GM and the scores from the four cognitive components we performed behavioral PLS analysis. Importantly, previous studies related SD_BOLD_ within spliced fixation periods in blocked fMRI series to performance on task [13,14], while the current study is the first application of resting state data in investigating behaviorally relevant SD_BOLD_. The behavioral PLS analysis begins with the correlation matrix between the individual scores on the four cognitive components and each voxel’s SD_BOLD_; correlations are calculated across subjects. Then, this matrix is decomposed via singular value decomposition. This decomposition produces orthogonal latent variables (LVs) that optimally represent relations between SD_BOLD_ in GM voxels and cognitive performance. Each LV contains a spatial pattern depicting the brain regions where the activity shows the strongest relation to performance. In this analysis, because we examined the association with four cognitive components, four outcome latent variables (LV) were possible. We predicted that if there are domain-specific patterns of optimal SD_BOLD_, then multiple LVs may be significant, each representing an association between a different cognitive construct and BOLD variability. Alternatively, if SD_BOLD_ is a more general feature common to different cognitive functions, there should be one LV representing the brain-performance relationship.

**Fig 1:**
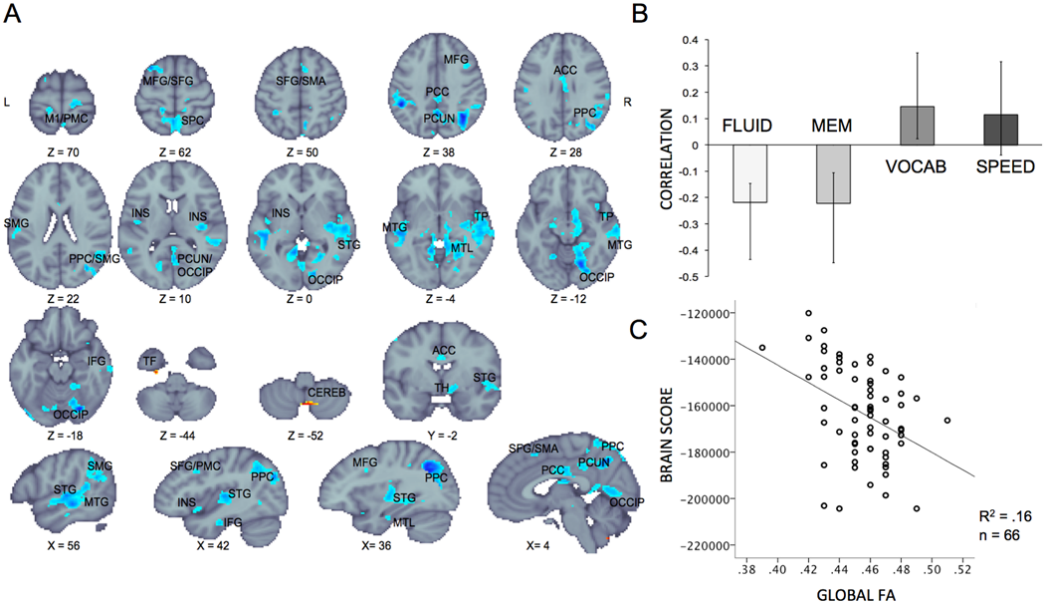
Multivariate relationships between cognitive performance and SD_BOLD_. **A**: PLS spatial pattern. Blue regions indicate greater and yellow/red regions indicate lesser SD_BOLD_ with better performance on fluid and memory, and worse performance on vocabulary. Significant regions: bootstrap ratio > ±3. M1: primary motor, PMC: premotor cortex, MFG: middle frontal gyrus, SFG: superior frontal gyrus, SMA: supplementary motor area, PCC: posterior cingulate gyrus, PCUN: precuneus, ACC: anterior cingulate cortex, PCC: posterior parietal cortex, SMG: supramarginal gyrus, INS: insula, OCCIP: occipital cortex, STG: superior temporal gyrus, TP: temporal pole, MTG: middle temporal gyrus, MTL: medial temporal lobe, IFG: interior temporal gyrus, TF: temporal fusiform, CEREB: cerebellum, TH: thalamus, **B**: Correlation magnitudes (Pearson r) between 4 cognitive constructs and SD_BOLD_ during rest (permuted p < 0.001, error bars represent bootstrapped 95% confidence intervals). The speed construct did not contribute to the LV as its error bars cross the zero. **C**: Scatterplot showing the relationship between global FA (WM integrity) and cognition–SD_BOLD_ relationship.

Our results supported the latter hypothesis: the PLS multivariate analysis yielded one significant latent variable (permuted p = 0.023, 59.46% cross-block covariance explained by this LV), suggesting that, overall, higher SD_BOLD_ was related to better performance on fluid and memory constructs and lower performance on vocabulary. This relationship was reversed in only two small clusters (Fig. 1A). The same analysis with additional controlling for the global signal (i.e. centering the mean across the volumes) yielded the same spatial pattern, where higher SD_BOLD_ was related to better performance on fluid and memory constructs (permuted p = 0.007, 60.41% cross-block covariance explained by this LV).

If the PLS model was run with vocabulary only (1 LV possible), only the clusters in temporal fusiform and cerebellum were above p <. 001 threshold, but the overall model was not significant. This suggests that the red-yellow cluster shown in Fig. 1A is attributable to the relationship with vocabulary. Similarly, a model with 4 cognitive constructs and additionally chronological age (5 LVs possible) explained only ca. 3% more of cross-block covariance than the four construct model from Fig. 1, and showed the same spatial pattern. This suggests that age is not driving the function-performance result from Fig. 1A. In this model age was positively related to vocabulary performance, but inversely to memory and fluid abilities, and SD_BOLD._

Perceptual speed did not significantly contribute to the observed performance–SD_BOLD_ correlation pattern, although there was a trend towards greater perceptual speed being related to lesser SD_BOLD._ Peak voxels’ location and bootstrap ratios are reported in Table 2.

**Table 2:** Significant clusters representing SD_BOLD_ and cognitive performance relationship.

Region	MNI coordinates (x, y, z)	BSR	p-value	Cluster size (voxels)
Fusiform/Visual	26, -76, -16	-6.12	0.0000	4313
Posterior parietal	36, -60, 42	-5.80	0.0000	1255
Inferior parietal lobule/SMG	-48, -42, 38	-5.63	0.0000	479
Precuneus	6, -60, 44	-5.09	0.0000	842
Lingual/V2	-10, -50, -2	-4.57	0.0000	492
MFG	-30, 16, 62	-4.53	0.0000	122
STG	-52, -26, -2	-4.46	0.0000	516
Occipital cortex	10, -86, 42	-3.89	0.0001	51
Cingulate (ant/post)	2, -16, 34	-3.86	0.0001	291
Lateral occipital (V4)	-46, -80, -18	-3.86	0.0001	20
Superior Thalamus/fornix	4, -16, 18	-3.85	0.0001	46
SFG/SMA	4, 20, 52	-3.83	0.0001	76
Temporal fusiform	-20, -56, -12	-3.82	0.0001	59
M1/premotor	14, -26, 70	-3.75	0.0002	46
Lingual/cerebellum	-10, -74, -20	-3.73	0.0002	79
SFG/MFG	-20, 4, 72	-3.66	0.0002	10
Inferior parietal	-34, -74, 32	-3.62	0.0003	14
Temporal lobe	42, -14, -28	-3.60	0.0003	149
Inferior parietal/SMG	-50, -22, 26	-3.59	0.0003	128
Superior parietal lobule	32, -38, 40	-3.54	0.0004	12
STG	-60, -12, -8	-3.54	0.0004	56
MTG	-54, -52, 10	-3.54	0.0004	44
MFG	40, 12, 36	-3.50	0.0005	51
Inferior parietal/SMG	62, -30, 32	-3.49	0.0005	56
Superior parietal/precuneus	14, -42, 62	-3.48	0.0005	30
Insula	40, 12, -12	-3.48	0.0005	34
Superior parietal	-12, -64, 60	-3.47	0.0005	21
Superior parietal/precuneus	0.0, -40, 56	-3.45	0.0006	23
Superior Thalamus/fornix	18, -30, 14	-3.44	0.0006	41
Temporal pole	38, 16, -22	-3.43	0.0006	19
MTG	46, -60, 0	-3.43	0.0006	32
Occipital	-4, -84, 42	-3.39	0.0007	16
Dentate gyrus	-24, -28, -4	-3.34	0.0008	28
Cerebellum	8, -36, -24	-3.34	0.0008	15
Precuneus/Parietal	22, -72, 28	-3.32	0.0009	27
Precuneus	12, -68, 26	-3.17	0.0015	10
Hippocampus cornu ammonis	-22, -14, -12	-3.17	0.0015	30
Cuneus/superior parietal	-16, -82, 32	-3.16	0.0016	10
Cerebellum	-2, -64, -52	3.42	0.0006	14
Temporal fusiform	-28, -16, -44	3.41	0.0006	11

All peaks and clusters were determined using a voxel extent ≥10, minimum distance 10mm, and bootstrap ratio (BSR) ≥ 3.00. MNI, Montreal Neurological Institute (mm).

### WM integrity predicts function-cognition relations independent of age

Next, we investigated whether the observed associations between memory and fluid performance and SD_BOLD_ are related to the integrity of structural connections in the brain. To examine this hypothesis, we first obtained a summary measure of each participant’s expression of the significant LV pattern by calculating “brain scores”. This involved multiplying each voxel’s weights from the significant LV by the SD_BOLD_ in that voxel for each person, and summing it over all brain voxels. Thus, in a single measure, a brain score indicates the degree to which a subject expresses the multivariate spatial pattern of performance–SD_BOLD_ associations reported in the LV depicted in Fig. 1 (see Methods for more details on brains score calculation). Specifically, a person with a higher brain score showed better performance on memory and fluid abilities and greater SD_BOLD_ in the voxels depicted in Fig. 1A.

Finally, we performed a multiple regression analysis with the brain scores as a dependent variable, age as the first independent variable and global FA (mean FA across the main WM tracts) as the second independent variable. Note that DTI data was available from only 66 out of 91. We included age in the model as both global FA (r = -.38 p = .002 n = 66) and brain scores (r = .21 p = .048, n = 91) were negatively related to age. In addition, memory was negatively related to age (see previous section). Therefore, it was important to test whether the SD_BOLD_–performance association is related to WM microstructure beyond the effects of chronological age. Indeed, we found that higher FA accounted for a significant amount of variance in brain scores, in addition to variance related to age (R^2^ Δ _age_ = 0.041, F c Δ _age_ = 2.77, df = 64/1, p-value = .101; R^2^ Δ _globalFA_ = 0.12. F Δ _globalFA_ = 8.7, df = 63/1, p-value = .004). We also note that global FA was not related to perceptual speed, memory and vocabulary components (p >. 50) and was related to fluid abilities only at a trend level (r = .23 p = .068, n = 66). Together, our results suggest that global WM integrity is associated with behaviorally relevant variability in the BOLD signal, beyond the effects of age.

We run an additional PLS model including age, four behavioral scores, and global FA (n = 66). It yielded one LV (p = .005, cross block covariance explained of 63%), where greater FA and younger age was related to greater SD_BOLD_. Global FA contributed most to the relationship (r>0.4), and age to a lesser degree (r>0.2). Greater fluid intelligence and memory were also related to greater SD BOLD, but their contribution to the model was not significant (while vocabulary and processing speed showed a negative non-significant association). This result confirms that WM integrity is related to SD_BOLD_, that brain structure-function relationship may be stronger than brain-performance associations, and this issue should be further investigated (see Discussion). We highlight, however, that the purpose of this article was to investigate the structural WM correlates of behaviorally relevant SD_BOLD_ only.

## Discussion

We investigated the associations between resting SD_BOLD_ and performance on four distinct cognitive constructs in healthy older adults with a whole-brain, multivariate approach. We demonstrated that 1) better fluid abilities and memory was linked to greater SD_BOLD_ in multiple regions including precuneus, insula, temporal, parietal, and prefrontal regions, and cingulate, and 2) behaviorally relevant SD_BOLD_ pattern was shared by fluid abilities and memory. Moreover, inter-individual differences in these SD_BOLD_-cognition relationships were related to the global WM integrity, above and beyond the effects of chronological age.

### Association of SD_BOLD_ with performance differs by cognitive domain

A previous study reported that greater SD_BOLD_ in healthy adults was associated with younger age, faster, and more consistent response times (RT) across three levels of a perceptual match-to-sample task (immediate comparison, cued short-delay comparison, and delayed comparison; [14]). Our results provide further evidence for greater SD_BOLD_ being related to better performance in aging. Specifically, we showed that the cognitive constructs requiring adaptive and flexible processing–fluid abilities and memory–were driving this positive SD_BOLD_–performance association. For example, tasks defining the fluid abilities require abstract reasoning and problem solving that enable optimal adaptation to a changing and complex environment [49]. Similarly, episodic memory involves association formation and binding, as well as flexible and context-dependent retrieval. As a result, both fluid abilities and memory should benefit from greater dynamic range and the ability to explore different network states at the neuronal level [4,12,50].

On the contrary, the vocabulary construct representing semantic knowledge requires robust retrieval of information from long-term memory that was acquired, stored, and reinforced over years. Thus, vocabulary knowledge operates on “hard-wired”, automatic and repetitive responses and therefore may benefit from less SD_BOLD_ at the neural level. As an additional behavioral PLS analysis with only vocabulary construct did not yield a significant LV, this result relating lower SD_BOLD_ to better vocabulary performance should be treated as preliminary and further investigated with more cognitive tasks defining this domain.

The dissociation of SD_BOLD_–performance relationship between the cognitive domains parallels their differential sensitivity to age. Namely, advanced age is related to decline in fluid abilities, memory and speed, with relative sparing of vocabulary knowledge [51,52]. The regions where we observed an association of SD_BOLD_ with fluid abilities and memory (visual cortex, temporal pole, insula, cingulate, parietal cortex, lateral frontal regions) overlap with regions showing decreased SD_BOLD_ in older compared to younger adults [13]. Therefore, we speculate that SD_BOLD_ might be one of the neural correlates underlying the discrepancy of age-related effects on the four main cognitive domains. Further exploration of this claim should be done by extending analyses to samples with broader age range.

### Behaviorally relevant SD BOLD may support integration of brain networks

Many regions where we observed a positive association of SD_BOLD_ with fluid abilities and memory have been defined as degree-based hubs, “rich club” regions, or connector hubs in structural and functional network analyses: posterior cingulate cortex, superior frontal, parietal and insular cortex, as well as inferior temporal and fusiform cortex [22,53–55]. Brain “hubs” are regions with high connectivity degree in a given neural community [23,55,56], while “rich-club” regions are the high-degree hubs that tend to connect to each other [57]. Of particular relevance to our findings are the connector hubs: regions highly connected primarily to distinct brain networks [58–60]. Such connector hubs are localized to the insula, parietal, premotor, lateral occipital, and dorsal superior frontal cortex [60], where we also observed higher SD_BOLD_ in better performing older adults. Connector hubs integrate functionally segregated domains with possibly very distinct processing or oscillatory properties. We therefore suggest that the hub’s high connectivity with multiple brain functional networks requires or results in the higher moment-to-moment variability in neural function, which should be reflected by greater SD_BOLD_. Importantly, we predict that such SD_BOLD_ related to a region’s cross-talk between different neural networks should be driven by high variability in signal frequency and not only by variability as a result of high amplitude signal with a constant frequency. Clearly, our results need to be followed by a direct comparison of SD_BOLD_ patterns with functional connectivity network properties, time-frequency analyses to tease apart time-constant SD_BOLD_ from time-varying SD_BOLD_, as well as changes in SD_BOLD_ and power-law exponents in fMRI signal between rest and task states [61,62], and their significance for cognitive performance in aging.

Despite careful removal of physiological noise with ICA, we acknowledge that some of SD_BOLD_ regions, such as posterior cingulate, occipital cortex and regions near large vessels such as temporal pole and regions along the brain midline, may partly overlap with respiratory or cardiac-related fluctuations [63,64]. High static cerebral blood flow (CBF) and high amplitude of low-frequency fluctuations in CBF at rest in regions such as posterior cingulate cortex and insula, however, suggest that spontaneous fluctuations of fMRI signal in these regions are neuronally-driven rather than of vasomotor origin [65].

Finally, we note that our analysis yielded one model for memory and fluid abilities instead of two LVs specific for each cognitive construct. This further supports the possibility that the hub-related pattern of greater SD_BOLD_ represents a common rather than a domain-specific neural feature. In other words, our findings suggest that preserving high SD_BOLD_ in regions associated with intra- and inter-network communication is linked with better performance on a set of cognitive tasks requiring flexible neural processing. We speculate that hub regions that show greater SD_BOLD_ during spontaneous brain activity at rest would also have the capacity for increased neural processing complexity during cognitive tasks (e.g. memory and reasoning) [5,62]. Longitudinal designs and broader age ranges should help to tease apart age-related from individual differences in SD_BOLD_.

### White matter as a scaffold for behaviorally relevant SD_BOLD_

Our study provided the first evidence for an association between whole-brain behaviorally relevant variability in the BOLD signal and WM integrity. Therefore, our study further extends previous reports on a positive relationship between WM integrity and task-related changes in BOLD signal [27,66], and structure-function brain network properties [54,67,68]. We propose that poor WM integrity, most likely due to age-related changes in myelination, precludes fast and reliable signal transduction. Consequently, optimal interaction between brain hubs within or between brain networks becomes impaired [69,70]. For instance, some signals may be “lost” in between the GM regions, others may not arrive in a timely fashion to be optimally integrated in the neural processes [27,71], or the resting kinetic energy of the system may not be sufficient to adjust to externally driven cognitive challenges [5,62]. This may result in reduced processing complexity that could be detected as reduced SD_BOLD_ at rest and during task, for instance, during the creation of mnemonic representations or updating information during mental rotation.

Our result that older adults with greater FA in all major WM tracts had greater SD_BOLD_ and better performance on memory and fluid abilities converges with previous reports on relationships between diffusivity properties and fluid intelligence defined by reasoning abilities, cognitive flexibility, episodic memory, and processing speed in older adults [25,72–75]; for a review see also [76]. Although we observed only a trend relationship between WM integrity and fluid abilities, this lack of strong diffusion-cognition association may be because our participants represented a relatively narrow age range and being relatively high functioning, healthy older adults (all qualified for the MRI, aerobic capacity test and an exercise intervention), which may limit the variability in the FA and behavioral measures.

Together, our data suggests that magnitude and spatial pattern of SD_BOLD_ that is linked to high cognitive performance–and therefore represents optimal complexity of neural processing–relies on the integrity of structural brain connectivity via WM in the healthy aging brain. Our findings lay foundation for future investigations addressing more specific questions about structural correlates of SD_BOLD_. One direction will be to define the regional (both GM and WM) specificity of WM- SD_BOLD_ associations in aging and across lifespan. Another important issue that needs to be addressed is the role of cortical atrophy and the related partial volume effect in estimating SD_BOLD_ in aging population, and the possible mediating role of GM volume on the SD_BOLD_–cognition associations.

## Conclusions

We found that greater SD_BOLD_ in multiple brain regions, most of which have been identified as inter- or intra-network connecting hubs, was linked to better fluid abilities and memory. This suggests that optimal dynamic range of neural processing in hub regions may support cognitive operations that specifically rely on moment-to-moment processing adaptability and flexibility. Moreover, we showed that this behaviorally relevant SD_BOLD_ is supported by global WM integrity. We conclude that SD_BOLD_ is a promising functional neural correlate of individual differences in cognition in healthy older adults.

## Supporting Information

S1 Dataset Demographic, DTI, cognitive, and brain score data for the 91 participants. “Win” in the variable name indicates this variable was winsorized.
(XLSX)
